# Electromagnetic Fields: Lai’s Response

**Published:** 2004-09

**Authors:** Henry Lai

**Affiliations:** Department of Bioengineering, University of Washington, Seattle, Washington, E-mail: hlai@u.washington.edu

I agree with Stevens that free radicals and changes in oxidative state in cells could play an important mediating role in some biological effects of nonionizing electromagnetic fields (EMFs), such as DNA damage. Certainly, cellular iron metabolism affects these processes. Genetic damage in cells can lead to malignancy and cancer. However, excessive cumulative genetic damages could also result in cell death. One possibility is that EMFs may be useful in treating cancer.

